# Progressive Transformation of Germinal Centers: A Systematic Review

**DOI:** 10.7759/cureus.104275

**Published:** 2026-02-26

**Authors:** Elias Keyrouz, Myriam Boueri, Tatiana Akl, Mia Harb, Anna Karena Issa, Rami Saade

**Affiliations:** 1 Otolaryngology - Head and Neck Surgery, Lebanese American University (LAU) Medical Center - Rizk Hospital, Beirut, LBN; 2 Division of Medical Genetics, Department of Pediatrics, Duke University Medical Center, Durham, USA; 3 Otolaryngology - Head and Neck Surgery, Gilbert and Rose-Marie Chagoury School of Medicine, Lebanese American University (LAU), Byblos, LBN

**Keywords:** follicular hyperplasia, germinal center expansion, immunohistochemistry, lymph node pathology, lymphoid malignancies, nodular lymphocyte predominant hodgkin lymphoma, progressive transformation of germinal centers, ptgc, reactive lymphadenopathy, systematic review

## Abstract

Progressive transformation of germinal centers (PTGC) is a benign histopathological process, typically manifesting as persistent, painless lymphadenopathy. It is of clinical significance because of its ability to mimic or coexist with other lymphoproliferative disorders. This systematic review summarizes the currently available literature to outline the clinical, pathological, and diagnostic features of PTGC and to identify areas that require further investigations to guide future clinical practice.

## Introduction and background

Germinal centers (GCs) are pivotal microanatomical structures within secondary lymphoid organs where B cells undergo rapid proliferation, somatic hypermutation, and selection, ultimately generating high-affinity antibodies that are crucial for effective immune responses [1]. However, the regulated function and architecture of GCs can sometimes be disrupted, leading to a histopathological process known as the progressive transformation of germinal centers (PTGC) [2]. Although PTGC is regarded as a benign lymph node architectural alteration, it carries important clinical implications due to its potential to mimic or coexist with lymphoid neoplasms, thereby raising concern for malignancy during diagnostic evaluation.

PTGC was first described by Lennert and Muller-Hermelink in 1975 [2] as a phenomenon observed in follicular hyperplasia, whereby secondary follicles enlarge, resulting in a poorly defined distinction between the germinal center and surrounding mantle zone lymphocytes [2,3]. This transformation is characterized by the abnormal expansion of GCs, accompanied by distinct histological and immunophenotypic features [4]. Clinically, PTGC most commonly presents as painless cervical lymphadenopathy, frequently affecting adolescents and young adults, which further contributes to its diagnostic complexity.

PTGC has garnered increasing interest due to its unique pathological manifestations and potential clinical implications [4-6]. Although its exact etiology and pathogenesis remain incompletely understood, current research suggests that PTGC develops through a multistep process involving the migration of T cells and mantle zone B cells into the GC, ultimately disrupting its normal microarchitecture [5,6]. Pan-B-cell markers such as CD19, CD20, and CD79a are expressed by infiltrating B cells; however, these cells lack GC markers like CD10 and BCL6 [6]. This immunophenotypic profile is diagnostically important, as it helps distinguish PTGC from malignant lymphoid neoplasms that may exhibit overlapping architectural features. Moreover, the extent of GC transformation varies, depending on the immunogenicity of the inciting antigen and the degree of antigen trapping [6].

Despite the growing recognition of PTGC, the existing body of literature remains largely composed of isolated case reports, small case series, and single-institution experiences accumulated over several decades. Previous reviews have predominantly been narrative in nature and have not employed systematic methodologies to comprehensively identify and synthesize available evidence. Consequently, reported clinical characteristics, lymphoma association rates, diagnostic approaches, and surveillance recommendations remain heterogeneous and difficult to interpret. To date, no systematic review has attempted to pool the dispersed data across the published studies to provide a structured synthesis of PTGC’s clinical, pathological, and prognostic features, highlighting the need for a methodologically rigorous analysis.

Given PTGC’s clinical significance, increasing efforts have been made to refine its diagnostic criteria and therapeutic approaches [4]. This systematic review aims to synthesize and critically evaluate the existing literature on PTGC, integrating findings from a collection of pertinent studies. By assessing the current state of knowledge regarding PTGC's clinical presentation, histopathological characteristics, diagnostic modalities, management strategies, and potential prognostic factors, this review seeks to provide a comprehensive overview of PTGC and identify gaps in understanding that warrant further investigation.

## Review

Methods

Search Strategy

**Figure 1:**
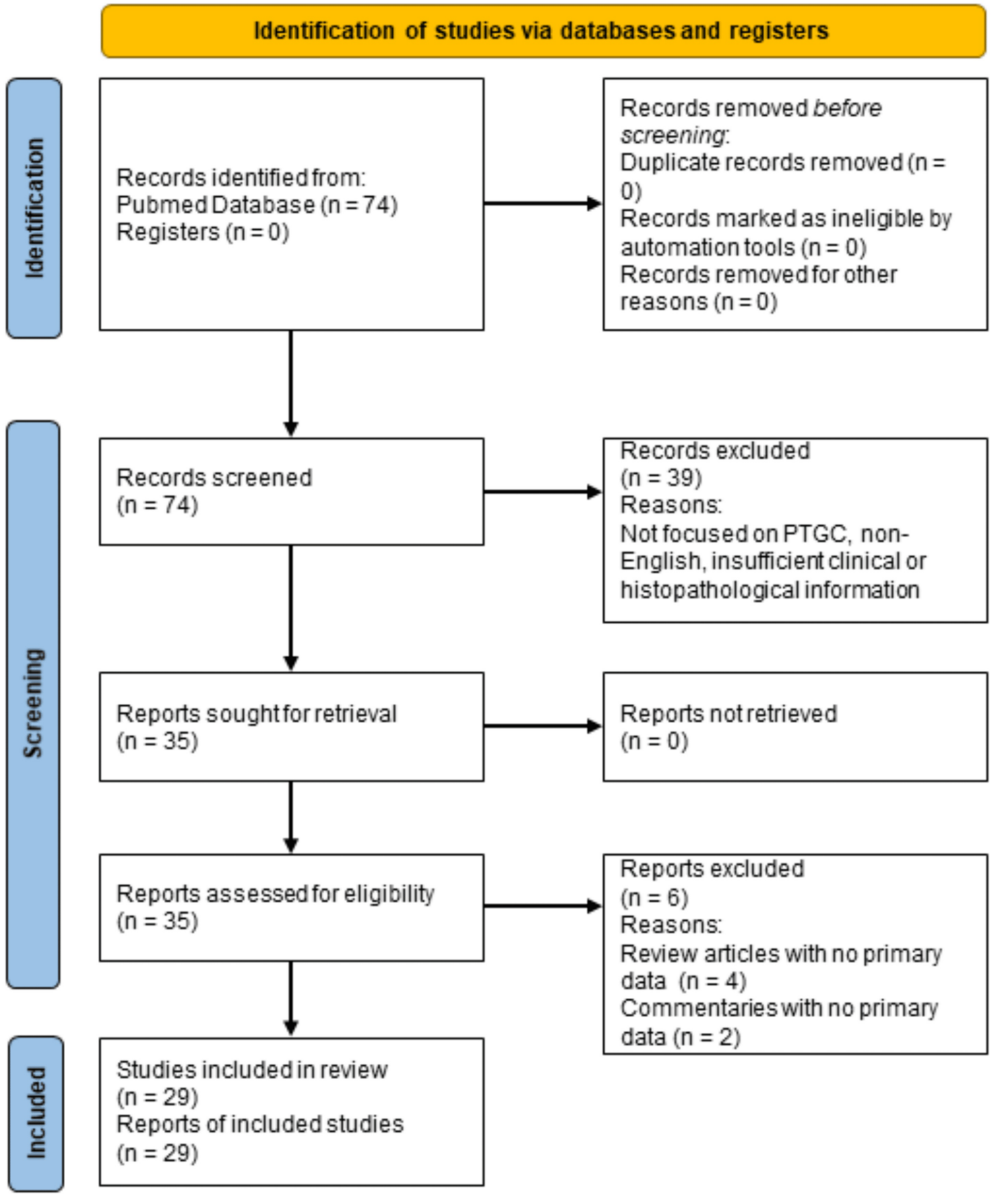
PRISMA flow diagram showing results of the database search *Abbreviations*: PRISMA: Preferred Reporting Items for Systematic Reviews and Meta-Analyses; PTGC: Progressive Transformation of Germinal Centers

Three investigators (E.K., M.B., and M.H.) conducted a systematic search of the PubMed database for studies reporting on PTGC, published from the database inception up to September 23, 2024. No other databases or registries were searched. A total of 74 articles were initially identified, and the titles and abstracts of these articles were screened. After screening, 39 articles were excluded because they were not focused on PTGC, lacked sufficient clinical or histopathological information, or were not published in English. The full texts of the remaining 35 articles were assessed, of which six studies (four review articles and two commentaries) were excluded from the systematic synthesis due to the absence of original patient data and were used only for background context. No language or study-type filters were applied in the database search; these criteria were applied during the screening stage. The complete PubMed search syntax, including search terms, filters, and date of search, is provided in a table in the Appendices. The study selection process is outlined in the Preferred Reporting Items for Systematic Reviews and Meta-Analyses (PRISMA) flow diagram (Figure 1).

PICOS Framework

The eligibility criteria were defined according to the PICOS framework (Table 1). This review focuses on a descriptive clinical and histopathological entity rather than an intervention and encompasses heterogeneous study types. Hence, the classical PICO (Population-Intervention-Comparison-Outcome) framework was not fully applicable and the PICOS (Population-Intervention-Comparison-Outcome-Study design) was adopted.

**Table 1:** PICOS framework *Abbreviations*: NLPHL: Nodular lymphocyte-predominant Hodgkin lymphoma; PICOS: Population, Intervention, Comparison, Outcome, Study Designs; PTGC: progressive transformation of germinal centers.

PICOS Element	Description
Population (P)	Patients of any age with PTGC, confirmed clinically and/or by histopathology
Intervention/Exposure (I)	Not applicable (descriptive systematic review without interventions)
Comparison (C)	Not applicable (no comparator groups included)
Outcomes (O)	Clinical presentation, histopathological characteristics, diagnostic modalities, associated conditions (e.g., NLPHL, autoimmune disease), management approaches, and reported outcomes including recurrence or progression
Study Designs (S)	Case reports, case series, and retrospective observational studies, prospective cohort. Review articles and commentaries were excluded from synthesis.

Inclusion and Exclusion Criteria

Titles and abstracts of the retrieved articles were screened for eligibility based on predefined criteria. Studies were included if they (a) were peer-reviewed articles available in PubMed; (b) primarily focused on PTGC; and (c) involved human subjects. A range of study designs, which included case reports, case series, retrospective observational studies, and one prospective cohort study, was considered. Studies (a) not published in English and (b) lacking substantial information on the clinical, histopathological, diagnostic, treatment, or prognostic aspects of PTGC were excluded. Review articles and commentaries were screened and used for relevant references and background information but were excluded from the synthesis due to the absence of primary patient data.

Study Selection Process

The selection process consisted of two stages: an initial title/abstract screening followed by a full-text review, both of which were conducted independently by the three investigators (E.K., M.B., and M.H.). Of the 74 retrieved articles, 39 were excluded because they were not focused on PTGC, lacked sufficient clinical or histopathological information or were not published in English. The remaining 35 underwent detailed assessment, which resulted in the inclusion of 29 studies. To minimize duplicate case inclusion, studies were reviewed for overlapping institutions, study periods, and patient cohorts. When potential overlap was identified, the most comprehensive or recent dataset was retained to avoid double-counting. Any disagreements were resolved through discussion and consensus.

Data Extraction and Synthesis

Data from the included studies were extracted by the study investigators using a standardized form, capturing key details such as authors, publication year, clinical presentation, diagnostic methods, histopathological findings, treatment approaches, and outcomes. Extracted data were synthesized descriptively in a tabular format and summarized in Table 2, providing a comprehensive overview of the study characteristics and findings. In addition, data regarding PTGC’s association with lymphoma were separately extracted and tabulated (Table 3).

**Table 2:** Summary of the articles and cases reported on progressive transformation of germinal centers (PTGC) *Abbreviations*: ABVD: Adriamycin, Bleomycin, Vinblastine, Dacarbazine; ANA: Antinuclear Antibody; CBC: Complete Blood Count; CBCD: Complete Blood Count with Differential; CHL: Classical Hodgkin Lymphoma; CHOP: Cyclophosphamide, Doxorubicin, Vincristine, Prednisone; CMP: Comprehensive Metabolic Panel; COP: Cyclophosphamide, Oncovin (Vincristine), Prednisone; CT: Computed Tomography; CVID: Common Variable Immunodeficiency; CXR: Chest X-Ray; DLBCL: Diffuse Large B-Cell Lymphoma; DVT: Deep Vein Thrombosis; DM: Diabetes Mellitus; EBV: Epstein–Barr Virus; ESR: Erythrocyte Sedimentation Rate; F-18 FDG: Fluorine-18 Fluorodeoxyglucose; F:M:  Female-to-Male ratio; FNA: Fine-Needle Aspiration; FNAB: Fine-Needle Aspiration Biopsy; FL: Follicular Lymphoma; GC: Germinal Center; HAART: Highly Active Antiretroviral Therapy; HIV: Human Immunodeficiency Virus; HL: Hodgkin Lymphoma; Hx: History; IHC:  Immunohistochemistry; IgG4: Immunoglobulin G4; Ki-67: Proliferation Marker Protein Ki-67; LAD: Lymphadenopathy; L&H: Lymphocytic and Histiocytic variant of Hodgkin Lymphoma; LN: Lymph Node; M:F: Male-to-Female ratio; MC-CHL: Mixed Cellularity Classical Hodgkin Lymphoma; MRI: Magnetic Resonance Imaging; NLPHL: Nodular Lymphocyte-Predominant Hodgkin Lymphoma; NR: Not Reported; SUVmax: Maximum Standardized Uptake Value; PET: Positron Emission Tomography; PET/CT: Positron Emission Tomography–Computed Tomography; PTGC: Progressive Transformation of Germinal Centers; US: Ultrasound; BCL2: B-cell Lymphoma 2; BCL6: B-cell Lymphoma 6; CD10, CD20, CD21, CD79a: Cluster of Differentiation surface markers used in Immunohistochemistry; C-MOPP: Cyclophosphamide, Vincristine (Oncovin), Procarbazine, Prednisone.

Author and Year of Publication	Clinical Presentation	Histopathology	Epidemiology	Diagnostic workup	Management/ Follow-up	Outcome
Talasiewicz et al. (2018) [4] Case series	Patient 1: Painless submandibular and cervical adenopathy	Patient 1: Giant, irregular-shaped GC was seen	Patient 1: a 32-year-old male	Patient 1: US showed hypoechoic, enlarged nodes without hilum; FNAB was inconclusive; PET-CT showed increased uptake (SUVmax 8.9); biopsy revealed PTGC	Patient 1: Surveillance due to hx of neuroendocrine pancreatic cancer; rituximab prophylaxis considered based on PTGC and HL association	NR
	Patient 2: Painless mass in the submandibular region with an enlarged, round, well-shaped LN	Patient 2: Follicular lymphoid hyperplasia with PTGC; IHC revealed normal BCL2, BCL6, and CD10 expression	Patient 2: a 65-year-old female	Patient 2: US showed hypoechoic LN with vascularization; FNAB suggested reactive LAD; MRI showed contrast-enhancing submandibular LN; biopsy revealed PTGC	Patient 2: Preferred lesion removal over active surveillance; rituximab prophylaxis considered for managing PTGC with a hx of HL	
Shaikh et al. (2013) [5] Retrospective study	Slow-growing asymptomatic LAD	Enlarged LN follicles and expansion of mantle zone lymphocytes into adjacent GCs	Median age at diagnosis was 11.5 years with a 2:1 M:F ratio	PET avid LNs seen in all four patients with PTGC who had PET-CT scan done	15 patients (52%) had more than one LN biopsy; cumulative incidence of a second biopsy after a diagnosis of PTGC: 42.3% and 12.2% at four years	NLPHL most common associated lymphoma; PTGC recurred in 17%; 14% had PTGC associated with HL (one preceding by four years, two concurrent, one subsequent); moderate risk of repeated biopsies; risk of progression to lymphoma was low; immune disorders including SLE, Castleman disease, and ALPS occurred in 24% of patients with four additional patients showing immune dysregulation without a unifying diagnosis; two patients demonstrated spontaneous resolution of LAD; at median 2.8-year follow-up persistent LAD was present in 38% while the remaining had no further enlargement after resection
Bailey et al. (2017) [7] Case report (excluding the review of articles reported in this table already [5,8-17])	Right neck LAD and an apparent ipsilateral parotid mass accompanied by fatigue and weight loss, after four years free of symptoms following a previous unknown pathology requiring superficial ipsilateral parotidectomy and neck dissection	NR	39-year-old female	Multiple FNAs of the cervical LNs and parotid mass were undiagnostic; pathology from previous surgery described PTGC; biopsy of level II cervical LN, sparing the previously operated parotid space, revealed NLPHL adjacent to areas of PTGC; postoperative PET/CT showed hypermetabolic LAD of the right neck, parotid, and right inguinal LNs	Chemotherapy	Good response to treatment
Verma et al. (2002) [8] Case series	Patient 1: One-year hx of intermittent episodes of LAD, each lasting a few weeks	Patient 1: Follicular hyperplasia with some follicles showing invasion by mantle zone lymphocytes and monocytoid B-cell hyperplasia; immunoperoxidase stains demonstrated predominantly B cells in the germinal centers and T cells in the interfollicular zones	Patient 1: 21-year-old male	Patient 1: Biopsy of LN showed PTGC; CT scan chest and abdomen was negative for other LAD; repeat LN biopsy two years later showed persistent PTGC	Patient 1: No treatment, follow-up only	Patient 1: Three years of follow-up, persistent stable cervical LAD
	Patient 2: 11-year history of persistent axillary LAD with three negative LN biopsies	Patient 2: Follicular hyperplasia with some follicles showing invasion of the germinal centers by small mantle zone lymphocytes; in some areas, the germinal centers were largely replaced	Patient 2: 32-year-old female	Patient 2: Biopsy of LN showed PTGC; CT scan chest and abdomen was negative for other LAD; repeat LN biopsy one year later revealed persistent PTGC	Patient 2: No treatment, follow-up only	Patient 2: One year of follow-up, persistent stable disease
Licup et al. (2015) [9] Case series	Patient 1: Left-sided cervical adenopathy of two-year duration	GCs with fused and expanded mantles, reactive follicles, and CD30-positive immunostaining	2:3 M:F ratio and ages ranging from eight to 13 years	Biopsy of the associated LN showed PTGC	NR	No lymphoma reported
	Patient 2: Asymptomatic submandibular mass noted for eight months in an otherwise healthy young boy					
	Patient 3: Nine-month hx of a left submandibular mass					
	Patient 4: Four-month hx of a progressively enlarging left-sided submandibular mass					
	Patient 5: Seven-month hx of a right-sided submandibular mass					
Özkan et al. (2016) [10] Retrospective study	Patients presented with asymptomatic, localized, or generalized LAD predominantly in the neck and axillary area	Loss of a defined border between germinal centers and mantle zone lymphocytes with reactive follicular hyperplasia; immunophenotyping showed CD20 positivity, surface IgD expression, BCL2 negativity, and BCL6 positivity	Mostly young adults (median age 43.8 years) and a 1:1 M:F ratio	NR	At least one additional biopsy was required in approximately one-third of patients because of persistent or recurrent LAD; CD20 expression suggested that transformation to lymphoma might be preventable with rituximab	PTGCs may precede, follow, or be concurrent with lymphoma; 84.8% of patients (28/33) did not develop lymphoma during a median follow-up of 23 months; five patients had PTGC with concurrent or previous malignant lymphoma, including PTGC detected three years after DLBCL and T-cell-rich B-cell lymphoma and seven years after NLPHL in three patients
Kojima et al. (2003) [11] Retrospective study	Patients presented with head and neck LAD without systemic symptoms such as fever or weight loss; 31% had localized chronic inflammatory or autoimmune conditions at the time of LN biopsy, including chronic sialadenitis, chronic tonsillitis, infectious epidermal cysts, hyperthyroidism, and bronchial asthma	Small lymphocytes with variable numbers of centrocytes, centroblasts, and immunoblasts; CD20 positivity; mantle zone lymphocytes with remnants of germinal centers; reactive follicular hyperplasia	Median age was 53 years with 29% aged 60 years or older and a 3:1 M:F ratio	NR	Only one patient was treated with COP plus radiotherapy	No malignant lymphoma developed during a follow-up period of five months up to 238 months (median 27 months); among 38 patients with follow-up information, LAD recurred in four patients at 12, 30, 60, and 84 months; one patient died of HCC; the remaining 37 were disease-free at last follow-up
Chang et al. (2015) [12] Case report	Pulmonary embolism	LN showed increased numbers of variably sized, markedly enlarged follicles with GCs exhibiting irregular, blurred margins; CD79a staining demonstrated infiltration and accumulation of mantle zone lymphocytes within the larger follicles with BCL2 negativity, excluding follicular lymphoma; classical Reed–Sternberg–like cells were absent	67-year-old male	Serologic screening for coagulopathy, including antithrombin III, protein C, and protein S deficiencies, was negative; bilateral lower-limb duplex ultrasound showed no DVT; CT abdomen with contrast revealed a solitary 4.9×3.0 cm soft-tissue mass in the mid abdomen; F-18 FDG PET/CT demonstrated an intensely avid solitary mass; excisional biopsy with flow cytometry was compatible with PTGC	NR	NR
Burns et al. (1984) [13] Retrospective study	LAD	PTGC mimicking nodular lymphocyte and histiocyte–predominant HL with coexisting PTGC and L&H Hodgkin disease in 18% of cases; PTGC preceded L&H Hodgkin disease in two patients and followed it in three; neoplastic nodules contained dendritic reticulum cells and B lymphocytes with scattered T cells; differentiation from L&H Hodgkin disease was based on cellular composition and architecture	171 cases of nodular lymphocyte-predominant (L&H) Hodgkin’s disease in typically young to middle-aged adults	LN biopsies done; immunologic studies on frozen tissue sections showed B-cell-predominant nodules	NR	Need for surveillance in PTGC patients due to the risk of later lymphoma
Hansmann et al. (1990) [14] Retrospective study	Most LAD localized in the cervical region, followed by the inguinal and the axillary regions	PTGCs were larger than florid GCs and mainly composed of small lymphocytes; circumscribed areas of centroblasts or remnants of florid GCs were present within PTGCs; proliferation of small clusters of predominantly medium-sized Ki-67–positive cells in the mantle zone	Peak incidence in the fourth decade, with a male predominance (M:F=2.2:1)	NR	Relapses occurred in 14 of 66 cases within 20 days to 35 months; second PTGC developments occurred in the same LN region in 12 cases and in different regions in two cases	Among 66 cases, three patients (5%) developed HL over a follow-up of three months to seven years; four patients had coexistent HL, and four others had previous HL with subsequent PTGC occurring from a few months up to four years later
Ferry et al. (1992) [15] Case series	Patients presented with adenopathy involving several nodal groups in three patients and localized adenopathy in two; nodes affected cervical, inguinal, and axillary nodes measuring 3-4 cm	Nodal architecture was significantly distorted and suggestive of NLPHLD, but Reed–Sternberg cells were absent in three cases, consistent with PTGC	Mean age was 18 years with a range of 14-24 years	Excision of LNs showed PTGC counts per specimen ranging from 10 to 123 with a mean of 67; individual sections contained nine to 29 PTGCs, with a mean of 19	All patients were untreated and observed with follow-up only	Three patients had persistent adenopathy from one year and four months up to 10 years post diagnosis; repeat biopsy in two patients at two and three years post diagnosis showed florid PTGC without Hodgkin disease; one patient had a biopsy eight years after presentation showing only rare PTGCs; two patients with isolated LAD had no recurrent LAD at two and five years
Osborne and Butler (1984) [16] Retrospective study	Asymptomatic solitary enlarged LN	PTGCs measured three to four times the diameter of surrounding GCs and were composed predominantly of small round lymphocytes mixed with scattered immunoblasts and occasional benign histiocytes	Young males with a median age of 23	NR	NR	Among 50 PTGC cases, no patient developed HL during a 10-year follow-up; coexistent HL was present in 10% of cases; previous HL occurred in 30%; the largest group of 31 patients had no prior or subsequent HL; one patient developed multiple myeloma
Poppema et al. (1979) [17] Case series	Patient 1: Enlargement of left and right axillary LNs and right cervical LNs	PTGCs showed large size and high content of small lymphocytes compared with primary and secondary follicles; centroblasts, centrocytes, and dendritic reticulum cells were present and sometimes arranged in clusters; transitional forms between hyperplastic secondary follicles and progressively transformed GCs were observed; few residual starry-sky macrophages with phagocytosed lymphocytes were present; small groups of epithelioid cells were sometimes seen around PTGCs, while interfollicular areas remained uninvolved and contained typical elements (epithelioid venules, many lymphocytes, and a few reticulum cells)	Patient 1: 6-year-old male	Biopsy showed PTGC	NR	Patient 1: NR
	Patient 2: Enlargement of axillary and cervical LNs over 20 years before biopsy (treated only with radiation therapy)		Patient 2: 23-year-old male			Patient 2: Multiple recurrences over 30 years with diagnosis of NLPHL
	Patient 3: Enlargement of cervical LN		Patient 3: 21-year-old male			Patient 3: Later diagnosis of NLPHL
	Patient 4: Enlargement of axillary LN (with hx of NLPHL)		Patient 4: 32-year-old female			Patient 4: NR
	Patient 5: Enlargement of right axillary LN (with hx of NLPHL)		Patient 5: 36-year-old male			Patient 5: NR
Sadanand et al. (2023) [18] Retrospective study	Asymptomatic LN enlargement in the head and neck area	PTGCs showed expansion of mantle zone lymphocytes, follicular dendritic cells, and T cells into GCs; GCs were enlarged with disruption of normal architectural differentiation	Median age was 11 years with an almost 1:1 M:F ratio	CXR was most commonly done followed by US and CT scan of the neck and chest; frequently ordered labs included CBCD, CMP, ESR, LDH, and uric acid	Recurrent PTGC occurred in three of the 57 patients (5%) at intervals of three months, four months, and six years from the first biopsy	No patient developed a subsequent malignancy during the 20-year retrospective study period; concurrent NLPHL was diagnosed in three patients (5%), all treated with chemotherapy; three additional patients (5%) had a prior history of malignancy more than two years before PTGC diagnosis, including one with recurrent NLPHL, one with CHL, and one with T-cell/histiocyte-rich large B-cell lymphoma; one patient with prior CVID was managed at an outside center, another with refractory Evans syndrome was treated with steroids and rituximab, and an additional patient developed type 1 DM during the inclusion period
Wills et al. (2022) [19] Case report	Multiple enlarged LNs in the anterior cervical, supraclavicular, bilateral axillary, and bilateral groin regions with waxing and waning LAD	Large nodules were present in a background of follicular hyperplasia with PTGC lesions isolated and scattered among reactive lymphoid follicles, accompanied by follicular and interfollicular hyperplasia	2-year-old male	CBCD, EBV serology CXR, and US were done along with an excisional biopsy of the most prominent right axillary LN	Repeated PET/CT imaging and excisional biopsies were performed on three occasions over four years with follow-up every six months to assess the need for additional imaging or biopsy	No evidence of lymphoma
Vizcaino et al. (2018) [20] Case series	Patient 1: Right superolateral orbital lesion with right lacrimal gland enlargement in an HIV-positive patient on HAART	Patient 1: Lacrimal gland with patchy fibrosis, lymphoplasmacytic inflammation, enlarged lymphoid follicles, prominent mantle zones, and BCL2 and IgD positivity	Patient 1: 66-year-old female	Patient 1: MRI showed an enlarged lacrimal gland measuring 2.3 × 1.1 × 2.2 cm with low signal intensity on T1 and T2 and homogeneous contrast enhancement; incisional biopsy of the lacrimal gland and preorbital tissue with flow cytometry was negative for monoclonality	Patient 1: Treated with prednisone (0.6 mg/kg)	Patient 1: Near complete resolution of the lesion after two months of treatment
	Patient 2: Right lacrimal gland and orbital tissue lesion with a history of adenoid cystic carcinoma and Warthin tumor	Patient 2: Lacrimal gland with chronic inflammation, irregular, enlarged lymphoid follicles, prominent mantle zone, and BCL2 positivity	Patient 2: 71-year-old female	Patient 2: Incisional biopsy of the lacrimal gland and orbit was done	Patient 2: No treatment	Patient 2: Complete resolution after 10 months follow-up
	Patient 3: Bilateral eyelid swelling with recurrent left eyelid swelling	Patient 3: Fibroadipose tissue with scattered large polarized lymphoid follicles and a prominent mantle zone, focally penetrating germinal centers highlighted by BCL2 staining; interfollicular areas with plasma cells and small lymphocytes with sparse histiocytes and eosinophils; more than 100 IgG4-positive plasma cells per HPF	Patient 3: 55-year-old female	Patient 3: CT showed ill-defined enlargement of the left lacrimal gland; incisional biopsy of the lacrimal gland with PCR studies showed polyclonal IgG gene rearrangements	Patient 3: Steroids treatment	Patient 3: 12 years later, diagnosed with marginal B-cell lymphoma
Park et al. (2021) [21] Case report	Palpable left axillary LNs appearing six months after surgery for invasive ductal carcinoma of the breast	Follicular lymphoid hyperplasia with distended and occasionally coalescent GCs; CD3 and CD20 showed a reactive zonal pattern; CD21 and Ki-67 were positive within GCs; follicles were BCL2 negative	32-year-old female	Chest CT, breast MRI, and US demonstrated new axillary LNs with cortical thickening; PET/CT showed multiple hypermetabolic nodes in the left axilla, right neck, left external iliac region, and both inguinal regions; US-guided FNA was negative for malignancy; excisional biopsy was compatible with PTGC	No treatment, only four years of clinical imaging and follow-up	No interval changes in LN size, confirming a benign etiology
Makis et al. (2011) [22] Case report	Patient with DiGeorge syndrome presented with several weeks of upper respiratory tract infections, new red and black blisters in the head and neck region, pancytopenia, and splenomegaly	NR	14-year-old female	F-18 FDG PET/CT performed to rule out lymphoproliferative disease showed multiple enlarged FDG-avid lymph nodes in both axillae, the right inguinal region, and the right external iliac region; biopsy was compatible with PTGC	No treatment was administered, with LAD reassessed one year later by follow-up PET/CT	Complete resolution of axillary LNs with significant reduction in size and FDG uptake of the right inguinal and right external iliac nodes
Hod et al. (2023) [23] Case report	History of breast cancer with new painless axillary LAD	Enlarged LNs demonstrated follicular hyperplasia with large nodules and mantle zone lymphocytes extending into and disrupting the GCs; immunohistochemical findings were characteristic of PTGC	64-year-old female	PET/CT performed for breast cancer surveillance showed “false-positive FDG uptake” in two right axillary LNs concerning for malignancy; biopsy confirmed PTGC	NR	NR
Yashima et al. (2014) [24] Case report	Swelling of the submandibular LNs	Low-power view showed numerous PTGC areas with enlarged follicles lacking clear GC–mantle zone demarcation and containing predominantly small lymphocytes with scattered residual follicle center cells; high-power view demonstrated marked venular proliferation with enlarged endothelial nuclei and thickened basement membrane in interfollicular areas; Hodgkin and Reed–Sternberg cells were scattered in mantle and interfollicular zones; most small GC lymphocytes were positive for CD21, CD10, CD20, and CD79a with BCL2 negativity	60-year-old female	Thoracic CT demonstrated enlarged deep cervical and right axillary LNs, and biopsy revealed HL arising within PTGC	Three cycles of ABVD chemotherapy were administered, and relapse occurring nine months later in an axillary LN required an additional three cycles of ABVD	Complete remission
Sandhaus et al. (1988) [25] Case report	Enlarged right groin LN	Altered GCs contained a mixed population of small cleaved and large cleaved and noncleaved lymphocytes without the usual phagocytic histiocytes seen in benign follicles; mantle zones appeared irregularly discontinuous, producing a “floral” pattern that blended intrafollicular and interfollicular areas; lymphocytic composition of interfollicular zones was similar to follicular zones except for more numerous eosinophils and plasma cells; immunologic and molecular studies demonstrated lack of oligoclonality arguing against malignancy mimicking PTGC	55-year-old male	CXR, CBC, and chemistry profile were normal; LN biopsy demonstrated "floral variant" of follicular lymphoma, mimicking PTGC	NR	Six months after biopsy, no evidence of disease progression
Osborne and Butler (1987) [26] Case series	Patient 1: Abdominal mass	Patient 1: Relatively uniform round to oval lymphomatous nodules	Patient 1: 42-year-old male	Patient 1: Biopsy demonstrated follicular large-cell lymphoma	Patient 1: Chemotherapy with CHOP followed by C-MOPP after relapse, plus radiation therapy	Patient 1: Relapsed and was undergoing treatment again
	Patient 2: 9.0-cm left inguinal mass developed over a two-year period	Patient 2: Diffuse monomorphic proliferation of predominantly large noncleaved cells	Patient 2: 49-year-old male	Patient 2: Biopsy demonstrated diffuse large-cell lymphoma with negative staging laparotomy	Patient 2: Chemotherapy with CHOP and radiation therapy	Patient 2: Relapsed and was undergoing treatment again
	Patient 3: Midline cervical mass	Patient 3: Occasional large nodules measuring two to three times the diameter of background reactive follicles composed of small round lymphocytes admixed centrally with a few fragments of reactive GCs	Patient 3: 55-year-old female	Patient 3: Biopsy initially demonstrated PTGC and was later diagnosed as the “floral” variant of follicular large-cell lymphoma	Patient 3: Observation	Patient 3: NR
Handa et al. (2009) [27] Case report	Two-year history of slowly growing swelling in the right cervical region without fever	Numerous lymphoid follicles were present with GCs of varied size and shape clearly demarcated from mantle zones; GCs contained cleaved cells, transformed cells, and variable plasma cells; LN contained two PTGCs distinctly larger than adjacent GCs and composed predominantly of small lymphocytes with a few centrocytes, centroblasts, immunoblasts, and occasional plasma cells	39-year-old Japanese male	Biopsy confirmed PTGC, and PET imaging with repeat biopsy five years later was performed at LAD recurrence	Observation	Five years after initial diagnosis the patient developed recurrent LAD with biopsy showing non-Hodgkin lymphoma, treated with chemotherapy, leading to complete remission
Amin et al. (2012) [28] Case series	Three patients presented with orbital swelling and five with lacrimal gland involvement; one of the patients had a prior diagnosis of NLPHL	Four cases showed complete PTGC with enlarged GCs containing more than 90% mantle zone lymphocyte infiltration; three cases demonstrated incomplete PTGC with less than 90% infiltration; one case was reclassified as B-cell non-Hodgkin lymphoma; all cases demonstrated sequential reactive GC changes, including follicular hyperplasia, follicular lysis, and complete or incomplete PTGC, with morphology closely resembling PTGC patterns	Among eight patients, the F:M ratio was 2.5:1 with an age range of 24 to 73 years and a mean age of 54.4 years	Five biopsies were obtained from lacrimal glands and three from the orbital tissue	NR	NR
Miller et al. (2010) [29] Case report	One-month history of asymptomatic right cervical lymphadenopathy with a prior history of reactive lymphoid hyperplasia of the right orbit treated with radiation therapy 14 months earlier and a right preauricular mass on physical examination	Follicles showed preserved polarity with GC tingible body macrophages and flow cytometry demonstrated a polyclonal B-cell population; IHC highlighted mantle zone lymphocyte infiltration into GCs via BCL2 staining, and BCL6 labeling demonstrated altered centrocyte staining with non-labeled islands within PTGCs	75-year-old male	FNA of preauricular and cervical LNs demonstrated lymphoid hyperplasia; CT imaging showed multiple right cervical LNs without significant left-sided adenopathy, a 2.5-cm right parotid bed mass, and a residual right supraorbital mass; excisional biopsy of a right level two LN confirmed PTGC	NR	Follow-up demonstrated unchanged persistent LAD
Osborne et al. (1992) [30] Retrospective study	23 patients, with most presenting with a solitary asymptomatic enlarged LN, most commonly cervical	Epithelioid histiocytic clusters (44% versus 0%) often rimming PTGCs in pediatric HD-unassociated cases	Mean age was 11.3 years with a median of 11 years (range 4 to 16 years) and a M:F ratio of 18:5	Biopsy of LN	NR	16/23 (70%) patients showed no progression to HL; one patient (4%) subsequently developed NLPHL; pediatric cases demonstrated a higher recurrence rate than adult cases of PTGC at 50% versus 23%
Mohan et al. (2016) [31] Case report	One-year history of a progressively enlarging right parotid mass with associated cervical LAD	Florid follicular lymphoid hyperplasia with few follicles demonstrating infiltration of the GC by small mature mantle zone lymphocytes	10-year-old female	CBC, ESR, ANA, and immunoglobulin levels were normal; CT showed enlarged right intra-parotid and cervical LNs; US-guided biopsy of the parotid mass demonstrated a reactive lymphoid process; excisional biopsy confirmed PTGC; PET/CT demonstrated increased uptake in the right parotid region, bilateral jugulodigastric chains, right supraclavicular region, right axilla, peripancreatic region, and bilateral inguinal nodes	No treatment was given with follow-up for two years	Two years later the patient was diagnosed with papillary thyroid carcinoma and remained in remission for three years after treatment
Picardi et al. (2011) [32] Prospective non-randomized interventional cohort study with historical control	All patients had a history of treated HL with histologic diagnosis of PTGC during follow-up	Group 1: 18 focal nodules, 30 floral nodules, and 29 cases with reactive follicular hyperplasia	Group 1: 28 males and 20 females with a median age of 36 years	NR, and the histologic diagnoses of HL and PTGC were confirmed by biopsy	Group 1: 48 patients received rituximab prophylaxis in addition to initial chemotherapy and radiation therapy	Group 1: One patient relapsed with FL
		Group 2: 20 focal nodules, 28 floral nodules, and 25 cases with reactive follicular hyperplasia	Group 2: 29 males and 19 females with a median age of 37 years		Group 2: 48 patients underwent observation only after initial chemotherapy and radiation therapy	Group 2: 13 patients had HL relapse
Hartmann et al. (2015) [33] Retrospective study	LAD	General enlargement of GC, thickened mantle zones, and loss of clear borders between GCs and mantle zones; five architectural patterns identified	Male predominance	NR	NR	Interval between initial lymphoma and subsequent PTGC ranged from one to 16 years, and among 160 PTGC cases 93 had no antecedent or subsequent lymphoma, 23 showed synchronous PTGC with NLPHL, and 44 had antecedent or subsequent lymphoma (including 20 patients with prior NLPHL, 14 with previous or subsequent CHL, and 10 with other lymphoma types such as follicular lymphoma or cutaneous T-cell lymphoma)
		Pattern 1: Scalloped mantle zones with pseudopapillary protrusions of GCs				
		Pattern 2: Incomplete septum-like infiltration of mantle zone cells into residual GCs				
		Pattern 3: GCs partially or fully dissected by mantle zone B-cell septa of variable thickness				
		Pattern 4: Extensive mantle zone B-cell invasion leaving only small remnants of GCs				
		Pattern 5: Complete obliteration of the GC				

Given the heterogeneity in study design, patient populations, follow-up duration, and outcome reporting across the included studies, formal quantitative pooling (meta-analysis or meta-regression) was not feasible. Accordingly, a structured narrative synthesis approach was applied. Extracted data were organized according to study design, demographic characteristics, clinical presentation, lymphoma association, and management strategies. The findings were then compared across studies to identify the recurring patterns and areas of variability. When differences were observed, they were interpreted in light of the study design, sample size, and duration of follow-up.

A study by Bailey et al. [7], which reported a new case and reviewed previous cases, overlapped with some articles in this study [5,8-17]. To avoid redundancy, these overlapping articles were excluded from Table 3, as they were already counted by Bailey et al.’s PTGC/lymphoma analysis.

**Table 3:** Summary of the literature concerning the association between PTGC and lymphoma *Abbreviations*: PTGC: Progressive Transformation of Germinal Centers; NLPHL: Nodular Lymphocyte-Predominant Hodgkin Lymphoma; CHL: Classical Hodgkin Lymphoma; NR: Not Reported; FL: Follicular Lymphoma; NSHL: Nodular Sclerosis Hodgkin Lymphoma; CD20: Cluster of Differentiation 20.

Study	Cases of PTGC (n)	Lymphoma diagnosis in total (n)	PTGC before lymphoma (n)	Concurrent (n)	Lymphoma before PTGC (n)	Timeline
Tałasiewicz et al. (2018)[4]	2	0	0	0	0	NR
Bailey et al. [7] (reviewed cases reported in literature from 1979 until 2017; those overlapping with the ones included in our study were excluded from this table [5,8-17])	276	70	10	43	17	Lymphoma succeeding PTGC: anywhere from less than one year up to 13 years
Sadanand et al. (2023)[18]	57	6	0	3	3 (1 with recurrent NLPHL, 1 with CHL, and 1 with T-cell/histiocyte-rich large B-cell lymphoma)	Prior history of malignancy two or more years before PTGC diagnosis; retrospective review of PTGC cases over 20 years
Wills et al. (2022) [19]	1	0	0	0	0	NR
Vizcaino et al. (2018)[20]	3	0	0	0	0	NR
Park et al. (2021)[21]	1	0	0	0	0	NR
Makis et al. (2011) [22]	1	0	0	0	0	NR
Hod et al. (2023) [23]	1	0	0	0	0	NR
Yashima-Abo et al. (2014) [24]	1	1	0	1 (CHL)	0	NR
Handa et al. (2009)[27]	1	1	1 (FL)	0	0	Five years after the PTGC diagnosis
Amin et al. (2012)[28]	4	1	0	0	1	Six years after the initial diagnosis of NLPHL
Miller et al. (2010)[29]	1	0	0	0	0	NR
Osborne et al. (1992)[30]	23	7	1	1	5 (4/5 NLPHL and 1/5 NSHL)	NR
Mohan et al. (2016)[31]	1	0	0	0	0	NR
Picardi et al. (2011)[32]	48	48	0	0	48 (12/26 with history of NLPHL; 10/66 with history of CD20 positive CHL; 26/238 with history of CHL)	Median of 24 months (range between 10 and 72 months) of initial HL diagnosis; retrospective review of cases over eight years
Hartmann et al. (2015) [33]	160	67	2 (CHL)	23	42 (12 cases of HL, 10 cases of follicular lymphoma or cutaneous T-cell lymphoma, rest NLPHL)	Interval between initial occurrence of lymphoma and development of PTGC ranged from one year up to 16 years (median of five years)
Total	581	201	14	71	398	NR

Protocol Registration

This systematic review was not registered in a public protocol repository such as PROSPERO. Nonetheless, all methodological components - including eligibility criteria, search strategy, screening procedures, and data extraction - were pre-specified and consistently followed by the research team.

Risk of Bias Consideration

Heterogeneous studies were included in our review and, therefore, a design-specific risk of bias tool was used for each study type. The Joanna Briggs Institute (JBI) Critical Appraisal Checklist for Case Series, the JBI Critical Appraisal Checklist for Cohort Studies, the JBI Critical Appraisal Checklist for Analytical Cross-Sectional Studies, and the Risk Of Bias In Non-randomized Studies of Interventions (ROBINS-I) tool were used for case series, retrospective cohort studies, retrospective analytical pathology cross-sectional studies, and the prospective non-randomized interventional cohort study, respectively. For case reports, methodological quality was assessed using the JBI Critical Appraisal Checklist for Case Reports, recognizing that this tool evaluates reporting quality and internal validity rather than risk of bias in the causal inference sense. An overall risk-of-bias judgment (low, moderate, high, or serious) was assigned to each included study based on JBI checklist responses and study design characteristics.

Ethical Considerations

The Institutional Review Board (IRB) approval was not required as this systematic review included only previously published data. 

Results

Study Characteristics

A total of 29 studies were included in the qualitative synthesis (Figure 1). Among these studies, there was one prospective non-randomized interventional cohort study with historical control, nine retrospective studies of which two were retrospective analytical cross-sectional pathology studies and seven retrospective cohort studies, 11 single case reports, and eight case series, summarized in Tables 2, 3 [4,5,7-33].

Risk of Bias Assessment 

The risk of bias assessments for the included studies are presented in Tables 4-8.

**Table 4:** Risk of bias assessment of the case series Risk of bias was assessed using the Joanna Briggs Institute (JBI) Critical Appraisal Checklist for Case Series. Items were rated as Yes, No, Unclear, or Not Applicable.

Study	Were there clear criteria for inclusion in the case series?	Was the condition measured in a standard, reliable way for all participants?	Were valid methods used for identification of the condition?	Was there consecutive inclusion of participants?	Was there complete inclusion of participants?	Were participant demographics clearly reported?	Was clinical information clearly reported?	Were outcomes or follow-up results clearly reported?	Was the site/setting clearly reported?	Was statistical analysis appropriate?
Tałasiewicz et al. (2018) [4]	Yes	Yes	Yes	Unclear	Unclear	Yes	Yes	No	Yes	Not applicable
Verma et al. (2002) [8]	Yes	Yes	Yes	Unclear	Unclear	Yes	Yes	Yes	Yes	Not applicable
Licup et al. (2006) [9]	Yes	Yes	Yes	Unclear	Unclear	Yes	Yes	Unclear	Yes	Not applicable
Ferry et al. (1992) [15]	Yes	Yes	Yes	Unclear	Unclear	Yes	Yes	Yes	Yes	Not applicable
Poppema et al. (1979) [17]	Yes	Yes	Yes	Unclear	Unclear	Yes	Yes	Yes	Yes	Not applicable
Vizcaino et al. (2018) [20]	Yes	Yes	Yes	Unclear	Unclear	Yes	Yes	Unclear	Yes	Not applicable
Osborne and Butler (1987) [26]	Yes	Yes	Yes	No	No	Yes	Yes	Yes	Yes	Not applicable
Amin et al. (2012) [28]	Yes	Yes	Yes	Yes	Unclear	Yes	Yes	Unclear	Yes	Not applicable

**Table 5:** Risk of bias assessment of the retrospective cohort studies Risk of bias was assessed using the Joanna Briggs Institute (JBI) Critical Appraisal Checklist for Cohort Series. Items were rated as Yes, No, Unclear, or Not Applicable.

Study	Were the two groups similar and recruited from the same population?	Were the exposures measured similarly to assign people to exposed and unexposed groups?	Was the exposure measured in a valid and reliable way?	Were confounding factors identified?	Were strategies to deal with confounding factors stated?	Were participants free of the outcome at the start of the study?	Were outcomes measured in a valid and reliable way?	Was follow-up time reported and sufficient?	Was follow-up complete, and if not, were reasons described?	Were strategies to address incomplete follow-up used?	Was appropriate statistical analysis used?
Shaikh et al. (2013) [5]	Not applicable	Not applicable	Yes	Yes	No	Yes	Yes	Yes	Unclear	No	Yes
Özkan et al. (2016) [10]	Not applicable	Not applicable	Yes	Yes	No	Yes	Yes	Yes	Unclear	No	Yes
Kojima et al. (2003) [11]	Not applicable	Not applicable	Yes	Yes	No	Yes	Yes	Yes	Unclear	No	Yes
Hansmann et al. (1990) [14]	Not applicable	Not applicable	Yes	Yes	No	Yes	Yes	Yes	Unclear	No	Yes
Osborne & Butler (1984) [16]	Not applicable	Not applicable	Yes	Yes	No	Yes	Yes	Yes	Unclear	No	Yes
Sadanand et al. (2023) [18]	Not applicable	Not applicable	Yes	Yes	No	Yes	Yes	Yes	Unclear	No	Yes
Osborne et al. (1992) [30]	Not applicable	Not applicable	Yes	Yes	No	Yes	Yes	Yes	Unclear	No	Yes

**Table 6:** Risk of bias assessment of the retrospective analytical cross-sectional pathology studies Risk of bias was assessed using the Joanna Briggs Institute (JBI) Critical Appraisal Checklist for Analytical Cross-Sectional Studies . Items were rated as Yes, No, Unclear, or Not Applicable.

Study	Were the criteria for inclusion in the sample clearly defined?	Were the study subjects and the setting described in detail?	Was the exposure measured in a valid and reliable way?	Were objective, standard criteria used for measurement of the condition?	Were confounding factors identified?	Were strategies to deal with confounding factors stated?	Were the outcomes measured in a valid and reliable way?	Was appropriate statistical analysis used?
Burns et al. (1984) [13]	Unclear	Yes	Not applicable	Yes	No	No	Yes	Unclear
Hartmann et al. (2015) [33]	Yes	Yes	Not applicable	Yes	No	No	Yes	Yes

**Table 7:** Risk of bias assessment of the prospective non-randomized study Risk of bias of the prospective non-randomized study was assessed using the the Risk Of Bias In Non-randomized Studies of Interventions (ROBINS-I) tool, evaluating bias across pre-intervention, intervention, and post-intervention domains.

Study	Bias due to confounding	Bias in selection of participants	Bias in classification of interventions	Bias due to deviations from intended interventions	Bias due to missing data	Bias in measurement of outcomes	Bias in selection of the reported result	Overall risk of bias
Picardi et al., 2011 [32]	Serious	Moderate	Low	Low	Low	Moderate	Low	Serious

**Table 8:** Methodological quality assessment of the case reports Methodological quality was assessed using the Joanna Briggs Institute (JBI) Critical Appraisal Checklist for Case Reports, which evaluates reporting quality and internal validity rather than risk of bias in the causal inference sense.

Study	Were patient demographics clearly described?	Was the patient’s history clearly described and presented as a timeline?	Was the clinical condition of the patient on presentation clearly described?	Were diagnostic tests or assessment methods and results clearly described?	Was the intervention(s) or treatment procedure(s) clearly described?	Was the post-intervention clinical condition clearly described?	Were adverse events or unanticipated events identified and described?	Does the case report provide takeaway lessons?
Bailey et al. (2017) [7]	Yes	Yes	Yes	Yes	Not applicable	Unclear	Not applicable	Yes
Chang et al.(2015) [12]	Yes	Unclear	Yes	Yes	Not applicable	Unclear	Not applicable	Yes
Wills et al. (2022) [19]	Yes	Yes	Yes	Yes	Not applicable	Yes	Not applicable	Yes
Park et al. (2021) [21]	Yes	Unclear	Yes	Yes	Not applicable	Unclear	Not applicable	Yes
Makis et al. (2011) [22]	Yes	Yes	Yes	Yes	Not applicable	Unclear	Not applicable	Yes
Hod et al. (2023) [23]	Yes	Unclear	Yes	Yes	Not applicable	Unclear	Not applicable	Yes
Yashima-Abo et al. (2014) [24]	Yes	Unclear	Yes	Yes	Yes	Yes	Unclear	Yes
Sandhaus et al. (1988) [25]	Yes	Unclear	Yes	Yes	Not applicable	Unclear	Not applicable	Yes
Handa et al. (2011) [27]	Yes	Yes	Yes	Yes	Not applicable	Yes	Unclear	Yes
Miller et al. (2010) [29]	Yes	Unclear	Yes	Yes	Not applicable	Unclear	Not applicable	Yes
Mohan et al. (2016) [31]	Yes	Yes	Yes	Yes	Yes	Yes	Unclear	Yes

**Table 9:** Overall risk of bias of the included studies The overall risk of bias was classified as low, moderate or high. The designation of “serious” risk of bias for the prospective non-randomized study reflects concerns related to confounding and non-randomized allocation inherent to its design, consistent with established bias assessment frameworks for interventional observational studies.

Study	Study type	Overall risk of bias
Talasiewicz et al (2018) [4]	Case series	Moderate
Shaikh et al. (2013) [5]	Retrospective study	Moderate
Bailey et al. (2017) [7]	Case report (excluding the review of articles reported in this table already [5,8–17])	Moderate
Verma et al. (2002) [8]	Case series	Moderate
Licup et al. (2006) [9]	Case series	Moderate
Özkan et al. (2016) [10]	Retrospective study	Moderate
Kojima et al. (2003) [11]	Retrospective study	Moderate
Chang et al. (2015) [12]	Case report	High
Burns et al. (1984) [13]	Retrospective study	Moderate-High
Hansmann et al. (1990) [14]	Retrospective study	Moderate
Ferry et al. (1992) [15]	Case series	Moderate
Osborne and Butler (1984) [16]	Retrospective study	Moderate
Poppema et al. (1979) [17]	Case series	High
Sadanand et al. (2023) [18]	Retrospective study	Moderate
Wills et al. (2022) [19]	Case report	Moderate
Vizcaino et al. (2018) [20]	Case series	Moderate
Park et al. (2021) [21]	Case report	High
Makis et al. (2011) [22]	Case report	High
Hod et al. (2023) [23]	Case report	High
Yashima-Abo et al. (2014) [24]	Case report	High
Sandhaus et al. (1988) [25]	Case report	High
Osborne and Butler (1987) [26]	Case series	High
Handa et al. (2009) [27]	Case report	High
Amin et al. (2012) [28]	Case series	Moderate
Miller et al. (2010) [29]	Case report	High
Osborne et al. (1992) [30]	Retrospective study	Moderate
Mohan et al. (2016) [31]	Case report	High
Picardi et al. (2011) [32]	Prospective non-randomized interventional cohort study with historical control	Serious
Hartmann et al. (2015) [33]	Retrospective study	Moderate

Epidemiology

The reported demographic characteristics of patients with PTGC vary across studies. The condition has been reported in both pediatric and adult populations [8-10,18]. Studies indicate a higher prevalence in men compared to women [9,19]. PTGC has also been described in patients with autoimmune diseases, such as systemic lupus erythematosus (SLE) [18].

Clinical Presentation

The clinical presentation of PTGC is variable and can overlap with other lymphoproliferative disorders [4,5,11]. Commonly reported symptoms include painless lymphadenopathy, often involving cervical and axillary regions [8,9,11,18,19]. Systemic symptoms such as fever, night sweats, and weight loss are rare and non-specific [11,18].

Histopathological Findings

Immunohistochemical analysis assists in differentiating PTGC from malignant conditions; infiltrating B cells are typically CD20-positive, BCL6-negative or weak, and BCL2-negative, while surrounding T cells express CD3 and CD57 [5,10,11].

Imaging Findings

Data regarding radiologic findings in PTGC are scarce. PET/CT scan may reveal increased fluorodeoxyglucose (FDG) uptake, occasionally leading to false-positive interpretations [4,5,12,19,21-23]. Furthermore, the reported standardized uptake values (SUVmax) are generally low [12]. Serial PET/CT scans may help monitor PTGC regression or progression [22].

Association with Lymphoma and Other Systemic Diseases

PTGC can occur before, concurrently with, or after the diagnosis of Hodgkin lymphoma (HL) [5,10]. Even more, PTGC shares histopathological features with nodular lymphocyte-predominant Hodgkin lymphoma (NLPHL), and some patients alternate between these diagnoses upon repeated biopsies. Approximately 25% of PTGC cases have been reported to be associated with NLPHL [7], with individual study rates ranging from 7% to 18% [8,13-16,29].

While PTGC is rarely associated with other malignancies, cases of diffuse large B-cell lymphoma (DLBCL), classic Hodgkin lymphoma (CHL), and T-cell/histiocyte-rich large B-cell lymphoma have been reported [10,18]. The prognosis of the associated lymphoma is generally favorable [5].

In pediatric patients, PTGC has been associated with autoimmune conditions such as SLE, Castleman disease, and autoimmune lymphoproliferative syndrome (ALPS) [5,18]. Some cases involve undiagnosed syndromes characterized by autoimmune cytopenia, splenomegaly, or immunologic abnormality [5,18].

Management and Surveillance Patterns

No consensus surveillance schedule or standardized indications for repeat biopsy were reported across the included studies. Several studies emphasize counseling patients regarding the risk of biopsy recurrence and the potential, albeit low, risk of lymphoma progression. While PTGC itself carries a low risk of developing NLPHL, this transformation occurs more frequently than in the general population [19]. Regular follow-ups every four to six months are recommended [19], though some authors suggest annual follow-ups.

Since PTGC-associated lymphadenopathy most often occurs in superficial sites, follow-up through physical examination alone may be sufficient [5,19]. Several authors highlight the importance of monitoring the site of prior PTGC, as subsequent lymphoma may arise in the same location [14]. Pediatric patients have a higher recurrence rate than adults (50% vs. 23%) [30].

A recent review reported that only a minority of PTGC patients (nine in total) were evaluated by a pediatric hematologist/oncologist (PHO) prior to biopsy. Most patients were diagnosed with PTGC after referral. Only one-quarter (9/36) of those not initially evaluated by a PHO had baseline laboratory investigations, and none of them received in-person counseling about PTGC’s risks and its association with lymphoma and autoimmune diseases [18].

Discussion

This systematic review synthesizes available evidence regarding the clinical behavior and oncologic associations of PTGC.

Histologically, PTGC is characterized by a marked enlargement of germinal centers with altered centrocytic and centroblastic composition compared to conventional follicles [34]. The GCs become enlarged and may display an altered cellular composition, with mantle-zone B cells migrating inward [4,6]. Such features have prompted a discussion about the neoplastic potential of PTGC and its relationship with other lymphoproliferative disorders [34].

Notably, PTGC has also been defined as one of the recognized histologic patterns of IgG4-related lymphadenopathy, specifically in older patients, where PTGC may be accompanied by increased IgG4-positive plasma cells, highlighting an important diagnostic overlap between PTGC and IgG4-related disease [35].

Although PTGC accounts for approximately 4% of biopsies performed for chronic, nonspecific lymphadenopathy [7], its clinical significance lies less in prevalence and more in its diagnostic overlap with lymphoma.

Pathologists play a crucial role in differentiating PTGC from lymphomas and other reactive lymphoid conditions. The most significant entities in the differential diagnosis include NLPHL and lymphocyte-rich classic Hodgkin lymphoma, as well as various low-grade B-cell lymphomas [8,10]. Given the frequent association of PTGC with autoimmune disorders, screening for autoimmune cytopenias may be considered in all patients with further investigations tailored to the patient's clinical presentation [6].

The literature demonstrates significant variability in the diagnostic approach for PTGC (Table 2). However, the tiered approach previously suggested by Yan et al. [6] may be considered to reduce the risk of missing associated malignancies or systemic diseases. Imaging studies have a limited role in PTGC diagnosis, as PTGC-associated lymphadenopathy may exhibit FDG uptake patterns similar to those seen in malignant lymph nodes [4,5,12,19,21-23].

Patients should undergo a comprehensive physical examination and baseline blood work. Advanced testing is required in patients with additional risk factors. Yan et al. suggested a two-tier investigation system [6]. Tier one includes evaluations for malignancy (lymphoma), autoimmune conditions (SLE, autoimmune thyroid disease, autoimmune cytopenias), lymphoproliferative disorders (autoimmune lymphoproliferative syndrome (ALPS), primary immunodeficiency (common variable immunodeficiency), Castleman disease, and IgG4-related disease [6,20]. Tier two includes tests for specific infections and HIV [6]. Routine laboratory tests such as complete blood counts (CBC) with differentials, comprehensive metabolic panels (CMP), erythrocyte sedimentation rates (ESR), lactate dehydrogenase (LDH), and uric acid are usually unremarkable in PTGC [18].

Across the included studies, reported rates of lymphoma association varied considerably, which may reflect differences in the study design, follow-up duration, and patient selection [8,10,14-16,29]. Although lymphoma was reported in a subset of patients up to 13 years following PTGC diagnosis, the overall transformation rates ranged from 0% to 15% [8,10,14-16,29]. Taken together, these findings suggest that PTGC carries a low but clinically meaningful risk of lymphoma development rather than representing a uniformly premalignant condition.

For PTGC management, rituximab prophylaxis has been shown to improve event-free survival in Hodgkin lymphoma (HL) patients in remission following induction chemoradiotherapy [4,32,36]. One case described PTGC preceding papillary thyroid carcinoma, suggesting a possible immune-surveillance defect [31,37]. Given the biological similarities between pediatric and adult HL, extrapolation of rituximab benefit has been suggested; however, evidence supporting its use in isolated PTGC remains limited [6]. However, the optimal surveillance schedule and indications for repeat biopsies remain unclear. Short-interval follow-ups every four to six months over the long term have been suggested in the literature [5,6,18]. Patients may benefit from counseling regarding the small but real risk (around 9%) of progression to lymphoma within 13 years, as well as the potential need for repeat biopsies. Literature suggests that up to one-third to one-half of patients may require a second biopsy approximately four years after the initial PTGC diagnosis [5]. Given that most subsequent lymphomas occur in superficial lymph nodes, surveillance may primarily rely on thorough physical examinations rather than imaging [5,6,19]. Repeat biopsy and PET/CT scan imaging may be considered on a case-by-case basis. Finally, patients may be advised to seek urgent medical attention if they develop recurrent lymphadenopathy or systemic symptoms. Referral to a pediatric hematology/oncology (PHO) specialist may be appropriate in selected PTGC patients, particularly those with additional risk factors or diagnostic uncertainty.

Current studies on PTGC are predominantly retrospective in design and limited by small sample sizes. To date, only 14 patients, with unknown demographic and clinical details, have developed lymphoma subsequent to PTGC. Moreover, the scarcity of well-characterized cases makes it challenging to identify specific risk factors for PTGC transformation. Hence, the ability to stratify PTGC patients based on risk is limited, underscoring the urgent need for multi-center prospective studies.

The majority of the included studies were retrospective case series, cohort analyses, or case reports and were judged to have moderate to high risk of bias due to potential selection bias, limited adjustment for confounding variables, and incomplete follow-up reporting. Accordingly, reported lymphoma association and progression rates should be interpreted with appropriate caution.

**Figure 2:**
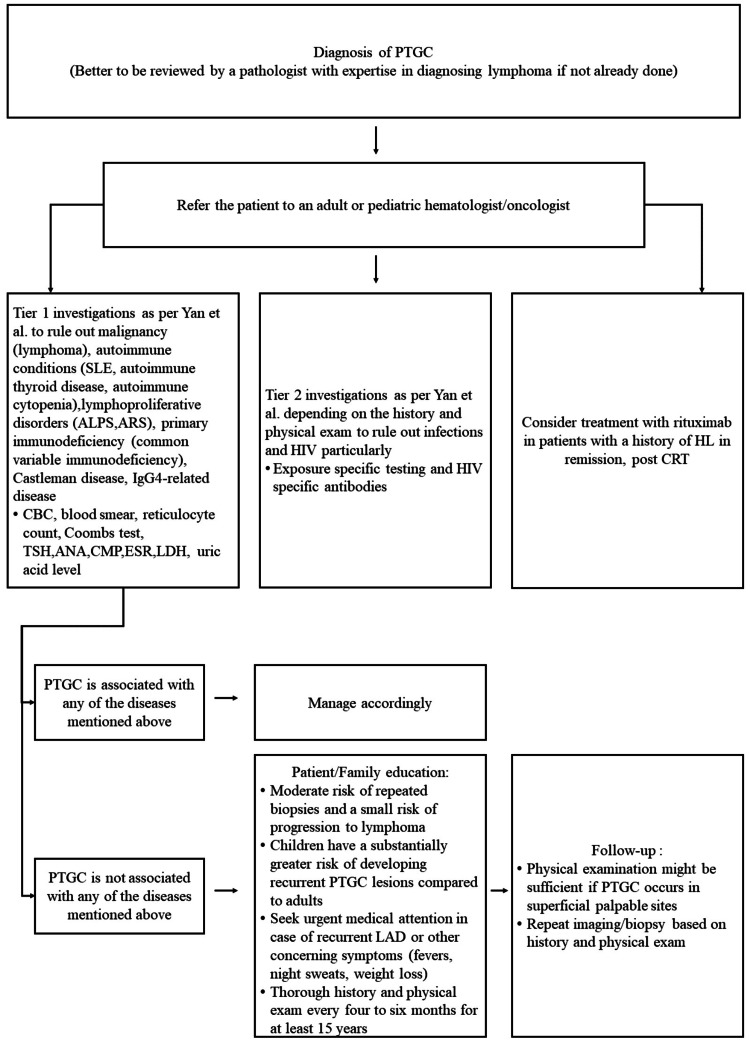
Algorithm for the diagnostic work-up and follow-up of patients diagnosed with progressive transformation of germinal centers (PTGC) *Abbreviations*: ALPS: Autoimmune Lymphoproliferative Syndrome; ANA: Antinuclear Antibody; ARS: ALPS-Related Syndromes; CBC: Complete Blood Count; CMP: Comprehensive Metabolic Panel; CRT: Corticosteroid Therapy; ESR: Erythrocyte Sedimentation Rate; HIV: Human Immunodeficiency Virus; HL: Hodgkin Lymphoma; IgG4: Immunoglobulin G4; LAD: Lymphadenopathy; LDH: Lactate Dehydrogenase; PTGC: Progressive Transformation of Germinal Centers; SLE: Systemic Lupus Erythematosus; TSH: Thyroid-Stimulating Hormone, The algorithm was created by the author based on concepts synthesized from the literature [6,14,30,32]. Image Credit: Elias Keyrouz.

To our knowledge, this review represents one of the most comprehensive syntheses of PTGC, encompassing its clinical presentation, epidemiology, diagnostic work-up, surveillance, and association with lymphoma and systemic diseases. Based on the findings of this review, an algorithm for the diagnostic evaluation and surveillance of patients with PTGC is presented (Figure 2).

Limitations

Many of the studies reviewed were retrospective in design, introducing potential biases and limiting the ability to establish definitive causal relationships. Additionally, the search strategy was limited to PubMed, which may have excluded studies indexed in other databases such as Embase, Scopus, or Web of Science, as well as certain gray literature sources. Although reference list screening was performed to identify potentially relevant studies, it remains possible that not all available evidence was captured. Moreover, small sample sizes and the heterogeneity in diagnostic criteria across the studies restrict the generalizability and consistency of the findings. The lack of comprehensive, long-term data hinders accurate assessment of the risk of lymphoma transformation and identification of specific risk factors for PTGC progression. Furthermore, management strategies for PTGC are not well standardized, and the absence of multi-center prospective studies impedes the development of definitive diagnostic, surveillance, and treatment guidelines. Lastly, the variability in clinical and demographic information, particularly in cases where lymphoma develops, makes it challenging to identify clear patterns or predictors of disease progression, highlighting the need for more rigorous investigations.

## Conclusions

PTGC is a rare but clinically significant condition characterized by abnormal expansion of GCs, often leading to diagnostic challenges due to its histologic overlap with various lymphoid disorders, including lymphoma. Although PTGC is frequently associated with autoimmune conditions, its potential progression to lymphoproliferative disorders, particularly NLPHL, necessitates close and prolonged monitoring. Current literature emphasizes the importance of accurate diagnosis through a combination of clinical, histopathological, and immunohistochemical evaluation. While the risk of PTGC transforming into lymphoma is low, it highlights the need for regular follow-up with physical examinations and, when necessary, repeated biopsies or imaging. Future prospective multicenter studies are essential to better understand the risk factors for lymphoma transformation and to refine management strategies. Ultimately, a multidisciplinary approach - involving hematology, oncology, and pathology specialists - is critical to ensure timely intervention and improve patient outcomes.

## Appendices

**Table 10:** Search strategy used

Database	Date	Search String	Filters Applied	Records Retrieved
PubMed	September 23, 2024	("progressive transformation of germinal centers"[Title/Abstract] OR "progressive transformation of germinal centres"[Title/Abstract] OR "PTGC"[Title/Abstract])	None	74
